# First Report on Development of Genome-Wide Microsatellite Markers for Stock (*Matthiola incana* L.)

**DOI:** 10.3390/plants12040748

**Published:** 2023-02-07

**Authors:** Chen Tan, Haimei Zhang, Haidong Chen, Miaotian Guan, Zhenzhi Zhu, Xueying Cao, Xianhong Ge, Bo Zhu, Daozong Chen

**Affiliations:** 1 College of Life Sciences, Gannan Normal University, Ganzhou 341000, China; 2 College of Plant Science and Technology, Huazhong Agricultural University, Wuhan 431700, China

**Keywords:** *Matthiola incana*, SSR, molecular makers, genetic diversity, genome sequencing

## Abstract

Stock (*Matthiola incana* (L.) R. Br.) is a famous annual ornamental plant with important ornamental and economic value. The lack of DNA molecular markers has limited genetic analysis, genome evolution, and marker-assisted selective breeding studies of *M. incana*. Therefore, more DNA markers are needed to support the further elucidation of the biology and genetics of *M. incana*. In this study, a high-quality genome of *M. incana* was initially assembled and a set of effective SSR primers was developed at the whole-genome level using genome data. A total of 45,612 loci of SSRs were identified; the di-nucleotide motifs were the most abundant (77.35%). In total, 43,540 primer pairs were designed, of which 300 were randomly selected for PCR validation, and as the success rate for amplification. In addition, 22 polymorphic SSR markers were used to analyze the genetic diversity of 40 stock varieties. Clustering analysis showed that all varieties could be divided into two clusters with a genetic distance of 0.68, which were highly consistent with their flower shape (potted or cut type). Moreover, we have verified that these SSR markers are effective and transferable within the Brassicaceae family. In this study, potential SSR molecular markers were successfully developed for 40 *M. incana* varieties using whole genome analysis, providing an important genetic tool for theoretical and applied research on *M. incana*.

## 1. Introduction

Stock (*Matthiola incana* (L.) R. Br.), an important annual ornamental plant, found worldwide, is a low-maintenance cool-season herbaceous biennial or perennial in the Brassicaceae family. As a well-known flower, stock cultivars with rich colors, a large number of flowers, prolonged flowering period, diverse flower shape, and various application scenarios, make it one of the few ornamental plants that can bloom in cool seasons [1,2,3]. In addition, *M. incana* is also an excellent oil and medicinal plant resource in the Brassicaceae family [4,5]. Unfortunately, the scientific research on *M. incana* is still relatively weak, and most reports focus on physiological characteristics [6,7], medicinal ingredients [5], flower color, or anthocyanin pathway genes [1,3], while research reports on the molecular level are still few and need to be strengthened.

Genetic analysis techniques based on DNA molecular markers can provide an efficient and accurate representation of a species’ genetic potential, regardless of age, physiological conditions, and environmental factors, thus helping to effectively characterize and utilize germplasm resources in breeding [8,9]. DNA molecular markers such as RFLP [10], RAPD [8,11,12], AFLP [13], ISSR [11,14] and SSR [8,12] are being widely used for genetic research, such as genetic mapping, marker-assisted selection breeding, and genetic diversity, etc., [15]. There are few reports on study using DNA molecular markers; among the limited reports, Dogan et al. (2016) used ISSR markers to study the phylogenetic relationship between Matthiola and other related genera [14]. Nakatsuka and Koishi (2018) developed specific codominant DNA molecular markers [16], and Ho et al. (2021) used RAPD and ISSR markers to identify double flowered plants [11]. The availability of more DNA markers will help improve our understanding of the genetics of *M. incana*.

Simple sequence repeats (SSRs), also known as microsatellites, are tandemly repeated DNA motifs occurring in both coding and noncoding regions [17,18]. It has been discovered that SSRs are ubiquitous in genomes, with repeat units of 1–6 nt. As the most useful DNA marker system for variety identification and germplasm management, SSR molecular markers have several advantages due to their abundance, high polymorphism, multiple alleles, co-dominance, low-cost, and the ease of assay by PCR [17,18], which have been applied extensively in genetic analysis, such as the analysis of population structure and genetic diversity [19,20], QTL mapping [21,22,23], marker-assisted selection breeding [24,25,26], and DNA fingerprinting [27,28], etc. SSR molecular markers are rarely used in *M. incana* [11,14], which is largely due to the fact that SSR molecular markers have not been developed in *M. incana*. Early development methods for SSR tagging suffered from cumbersome operations, low success rates and high-costs, or were limited by the information in public data. With the rapid development of next-generation sequencing (NGS) technologies, more and more species have been, or are being sequenced, which makes it possible to identify SSR loci at a result. Numerically accurate SSR loci, based on NGS techniques, can be developed quickly and cost-effectively. Thousands of SSR markers have been developed and applied in genetic studies in many species [17,18,29,30].

In the present work, we aim to identify and develop SSR molecular markers from the genome sequence of *M. incana*, based on the NGS date, and to use these markers to assess the genetic variation within stock cultivars. This research contributes to increasing the number of DNA molecular markers available for genetic studies of *M. incana*, which will lay the foundation for future breeding programs.

## 2. Result

### 2.1. Sequencing Data Statistics and Estimates of Genome Size and Heterozygosity

A total of 336 Gb clean reads were obtained, which accounted for 99.96% of the raw bases after filtering out low-throughput. The sequencing quality evaluation showed that Q30 was 93% (Table 1), which indicated that the high-throughput sequencing quality was good.

**Figure 1:**
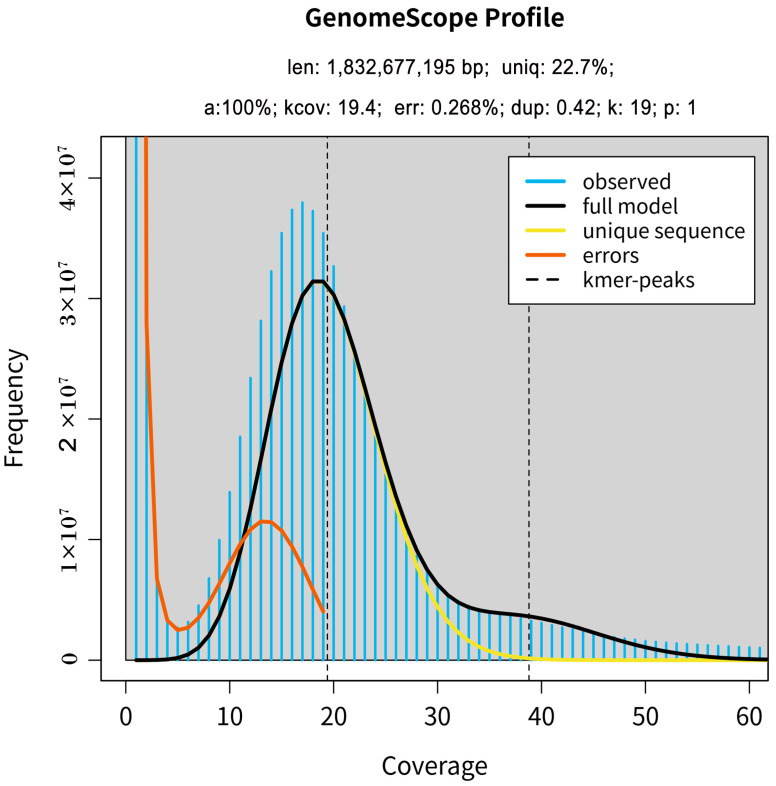
The distribution of K-mer = 17 depth. The estimated genome size = K-mer number/K-mer depth. The x-axis is depth; the y-axis represents the frequency at a particular depth divided by the total frequency of all depths.

For the estimation of genome size and heterozygosity, 17-mer was selected for K-mer analysis. In the 17-mer frequency distribution, the K-mer number was 38,360,865,911, and the K-mer depth was 17, so the initial estimate of genome size of *M. incana* is 1832.677 Mb (Figure 1). After excluding the effects of erroneous K-mers, the revised genome size is 1977.48 Mb (Table 1). Based on the K-mer map, a high peak (17) was observed at half the K-mer depth (17), which indicates that the *M. incana* genome has high homozygosity, and the heterozygosity rate is estimated to be 0.048%. In addition, the K-mer analysis and the repeat sequence rate was calculated to be 77.3%. The genome size of nonrepetitive sequences was estimated to be 416.02 Mb, which was approximately 22.7% of the *M. incana* genome (Figure 1). The low K-mer frequency indicated the error rate was 0.268% (Figure 1).

### 2.2. Preliminary Genome Assembly Results for M. incana

A total of 47.3 Gb of clean bases were used for preliminary genome assembly. The Hifiasm software was used to select the L parameter 2, which is the size of the assembled genome to be de-redundant, essentially half the size of the whole genome for diploidy results. The results show that a total of 891 contigs with a total length of 1,983,607,451 bp were obtained, with the longest assembled sequence being 41,745,843 bp and the length of N50 being 11,438,961 bp. For scaffolds, 343 scaffolds were obtained after further assembly, with a total length of 1,983,881,451 bp, and the maximum length and N50 length of the scaffolds were 357,731,900 and 258,639,456 bp (Table 1).

### 2.3. SSR Loci Identification of M. incana

The assembled data was used to look for SSR loci with a motif length of 2–6 bp. The motifs were grouped into di-nucleotides (Di-), tri-nucleotides (Tri-), tetra-nucleotides (Tetra-), penta-nucleotides (Penta-), and hexa-nucleotides (Hexa-). A total of 45,986 SSR loci were identified. The cumulative length of the SSR loci was 1,009,498 bp (Table 2), which comprises 0.05% of the assembled genome.

### 2.4. Characterization of SSRs

**Figure 2:**
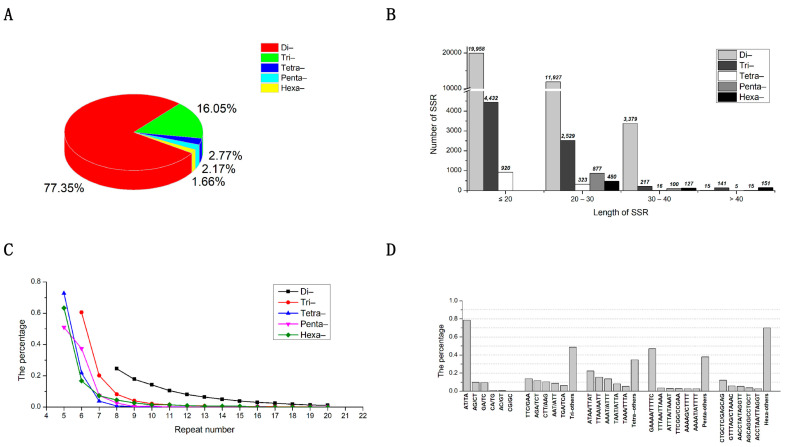
The characterization of SSRs identified in the genome of *M. incana*. (**A**) The frequency of SSR loci among SSR motifs (di- to hexa-nucleotides). (**B**) The distribution of SSR length from di- to hexa-nucleotide motifs. (**C**) The distribution of the repeat number of SSRs among SSR motifs (di- to hexa-nucleotides). (**D**) The distribution of major repeat motifs.

The frequency and density of the SSR loci in the *M. incana* genome decreased with increasing motif length (di- to hexa-nucleotides). That is to say, di-nucleotides (35,279; 77.35%) were the most frequently found types among the SSR motifs, and hexa-nucleotides (758; 1.66%) were the least frequently found (Figure 2A; Table 2). The SSR loci density was similar, with di-nucleotides having the highest density (17.84 SSR/Mb) and hexa-nucleotides having the lowest density (0.38 SSR/Mb) (Table 2). The length distribution of the SSR sequence ranged from 12–114 bp with an average of 21.95 bp. Most of the SSRs were less than 30 bp in length (Figure 2B). The general trend of changes in the length of SSR motif was that the number of SSRs gradually decreased with the increase of the length (Figure 2B). The average length of the repeated sequences was 21.76 bp, 20.70 bp, 21.49 bp, 28.56 bp, and 35.63 bp for repeat number; the number of SSRs decreased sharply as the number of repeats increased, especially those with larger repeat motifs. Tri- to hexa-nucleotide types had the biggest decrease, as repeats increased (Figure 2C).

In SSR loci, there were 481 types of repeat motifs (Table 2 and Table S2), and the number of motifs increased with increasing motif length (di- to hexa-nucleotides). The hexa-nucleotides repeat motifs (269) were the most abundant, but there were fewer SSR loci distributed in each type. The CTGCTC/GAGCAG (93) motif was the most widely distributed, accounting for just 12.27% of all the hexa-nucleotides repeat SSR motifs (Figure 2D; Table 2 and Table S2). The dominant di-nucleotides repeat unit was AT/TA, and the number of SSR sites was 27,723, which was the most frequent motif in the entire genome, accounting for 60.78% of the total SSR loci. Moreover, the base composition of SSR motifs was strongly biased toward A and T. The motif of TTC/GAA (13.76%), ATAA/TTAT (22.47%), and GAAAA/TTTTC (46.98%), had the highest frequency in tri- to penta-nucleotides motifs, respectively, (Figure 2D; Table 2 and Table S2).

### 2.5. The Distribution of SSR Loci on Chromosomes

**Figure 3:**
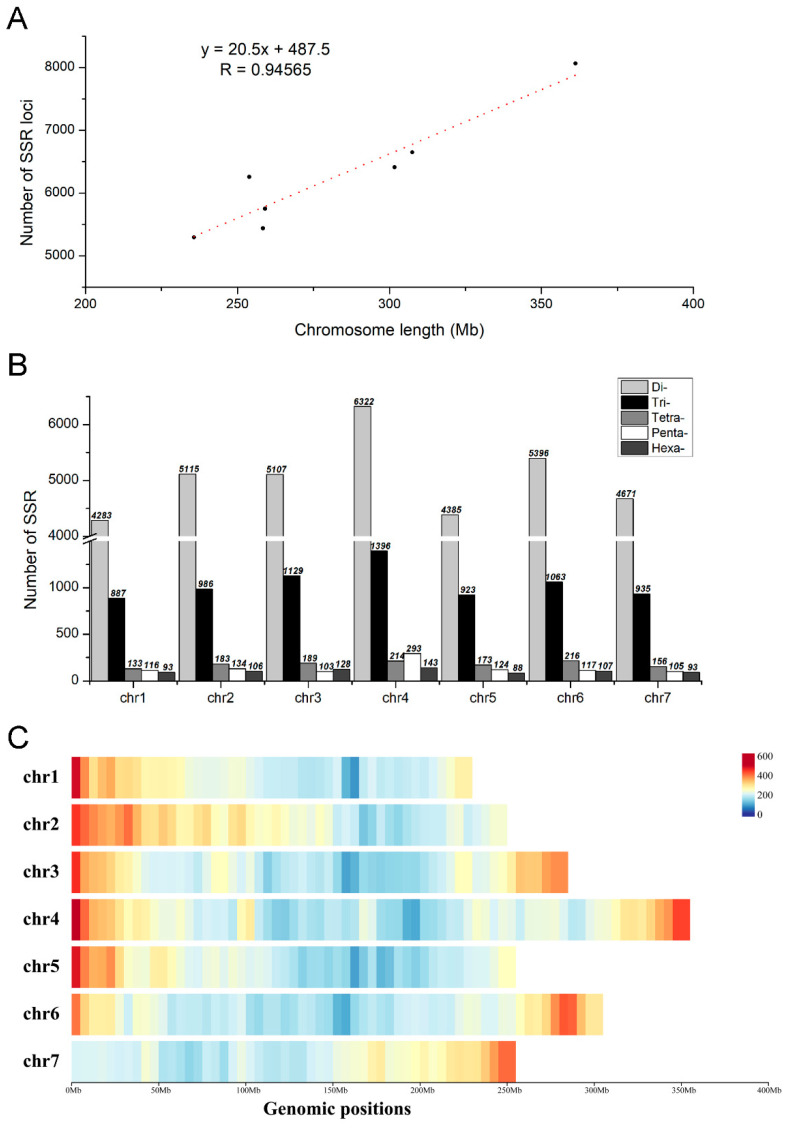
The distribution of SSRs on chromosomes. (**A**) Relationship between the SSR number and chromosome length. (**B**) The number of SSR loci on seven chromosomes of *M. incana*. (**C**) The density of SSR loci on seven chromosomes of *M. incana*; the bar represents the number of SSRs within a 10-Mb window.

The 45,612 SSR loci across seven chromosomes ranged from 5512 (chr1) to 8368 (chr4) (Table 3), and there was a significant positive correlation between the number of SSR loci and the chromosome length, with a correlation coefficient of 0.94565 (Figure 3A). However, the distribution of motifs of different length (di- to hexa-nucleotides) within a single chromosome was very similar to the pattern found throughout the whole genome. The most SSR loci were found in di-nucleotides repeat motifs, and the fewest in hexa-nucleotides repeat motifs; however, there were no big differences in SSR loci among chromosomes, which ranged from 22.03/Mb (chr5) to 25.69/Mb (chr2), with the average of 23.07/Mb (Figure 3B; Table 3). Furthermore, the distribution of SSR loci was similar on seven chromosomes, and the most abundant region was the chromosome arm, which was much higher than other regions (Figure 3C).

### 2.6. Genome-Wide SSR Primers Design and PCR Validation

Primer pairs were designed for 43,540 (95.46%), SSR loci (Table 3 and Table S3). Most of the SSR loci were di-nucleotides (33,308, accounted for 76.50%), followed by tri-nucleotides (7235, accounted for 16.62%) (Table 3), the average density of SSR loci, and the average distance between them. Among them, chromosome 4 had the highest number of SSR loci (8009) with a density of 22.17/Mb, while chromosome 1 had the lowest number of SSR loci (5263, 22.33/Mb) (Table 3).

**Figure 4:**
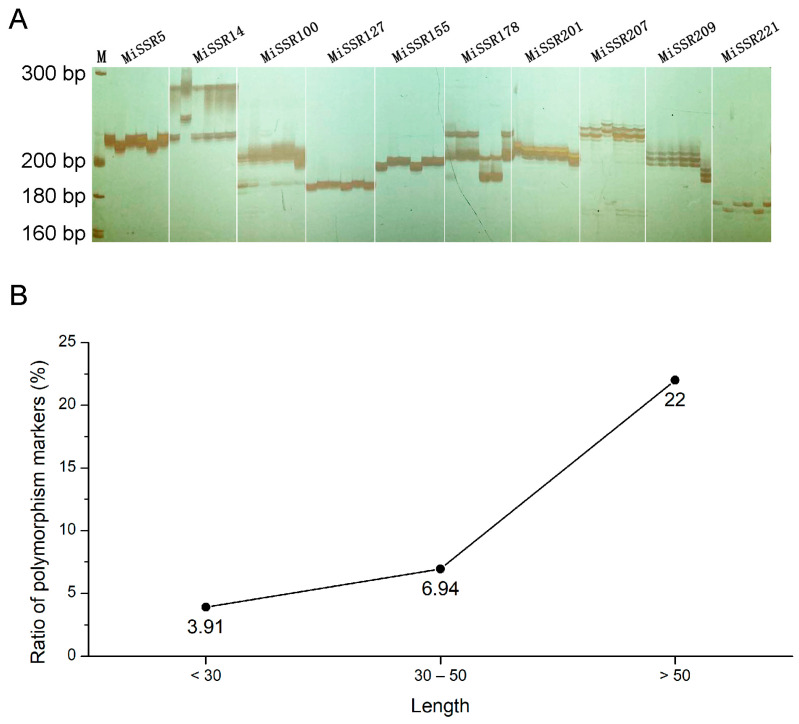
PCR verifies polymorphic markers. (**A**) The electrophoresis results of partial polymorphism markers in the six stock cultivars; M represents the DNA marker. (**B**) The ratio of polymorphic markers in SSR repeat motifs with different length.

In order to verify the effectiveness of primers in *M. incana*, 300 primer pairs were randomly selected for the di- to hexa-nucleotides’ motifs at SSR loci (Table S4). Genomic DNA from six different varieties of *M. incana* was used as a DNA template for PCR amplification. The results showed that among the 300 primer pairs, 40 exhibited unstable or no amplification, while the remaining 260 (86.67%) were effective, which were easy to identify, and repeatable (Figure 4A). Of these, 32 markers (10.67%) showed polymorphism in six different *M. incana* varieties (Table S4; Figure S1). The results show that the ratio of these polymorphic markers in the long sequence of SSR repeat motif increased significantly (Figure 4B). Only 3.91% of the markers in the SSR repeat motifs with lengths below 30 bp were polymorphic, and it increased to 6.94% in SSR repeat motifs with a length of 30–50 bp, while the ratio of polymorphic markers significantly increased in a longer sequence (larger than 50 bp) of SSR repeat motif, reaching 22.00% (Figure 4B).

### 2.7. Genetic Diversity Analysis of Stock Genotypes

Twenty-two markers distributed on seven chromosomes were used to analyze the genetic diversity of 40 stock cultivars. The phenotypic characteristics of these cultivars were also investigated in order to perform a correlation analysis of phenotypic features and genetic similarity (Table S1). A total of 70 bands were amplified for 22 SSR molecular markers from 40 cultivars, with an average of 3.2 bands per marker, and a variability range of 2–5. The observed number of alleles (Na), the effective number of alleles (Ne), and the Shannon’s information index (I) were calculated by the POPGENE software. The results showed that the average number of alleles (Na) was 3.0, the average number of effective alleles (Ne) per locus was 2.400, and the Shannon information index (I) was 0.886, indicating a rich diversity of these SSR molecular markers. Polymorphic information content (PIC) is an indicator of the diversity of loci, and the PIC of the 22 markers in this study ranges from 0.160 to 0.734, with an average of 0.525 (Table 4). Among them, 15 markers (68.18%) showed high polymorphism (PIC ≥ 0.5), and 5 markers showed moderate polymorphism (0.25 ≤ PIC < 0.5) (Table 4). These results indicate that the SSR molecular markers used in this study are highly polymorphic, and can be used for genotyping and genetic diversity studies.

**Figure 5:**
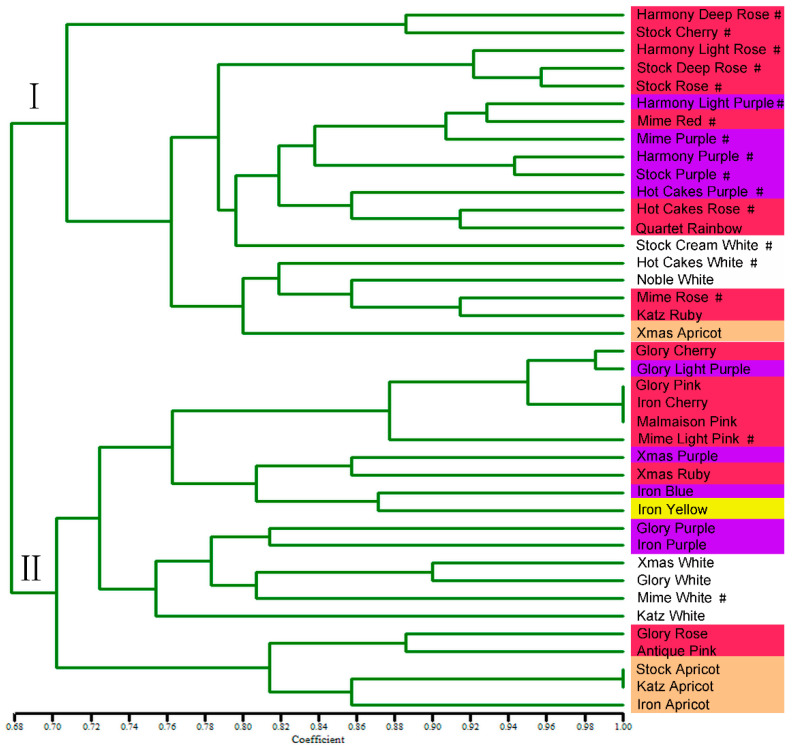
The UPGMA dendrogram of 40 stock cultivars based on 22 SSR markers. # represents potted types. Color bins represent flower colors, that is: the red bins represent deep red, rose, pink, or cherry flower; the purple bins are for deep purple and light purple flowers; the white bins represent white flowers; the yellow bins are for yellow flowers; and the orange bins represent apricot flower.

The genetic coefficients for the 40 tested cultivars ranged from 0.68 to 1.00 (Figure 5; Table S5), indicating that the genetic diversity was close among the cultivars. Of these, the two groups with a genetic coefficient of 1.00 were ‘Glory Pink’, ‘Iron Cherry’ and ‘Malmaison Pink’, and ‘Stock Apricot’ and ‘Katz Apricot’, respectively, indicating that the two groups are closely related. The dendrogram shows that stock varieties can be classified into two main clusters (I and II) with a genetic coefficient of 0.68. The first cluster (I) includes: all the ‘Harmony’ series cultivars; all the ‘Hot Cake’ series cultivars; most of the ‘Mime’ series cultivars (‘Mime Purple’, ‘Mime Rose’, and ‘Mime Red’); and some others are also included. Most of them were potted type, with lower plant height and more branches (Figure 5 and Figure S2A; Table S1). The second cluster (II) contains: all the ‘Glory’ series cultivars; all the ‘Iron’ series cultivars; most of the ‘Xmas’ series cultivars, except for ‘Xmas Apricot’; and some others (such as ‘Stock Apricot’, ‘Malmaison Pink’, and ‘Antique pink’) are also included. Most of them were cut-flower type, with higher plant height and no branches (Figure 5 and Figure S2A; Table S1). The clustering results indicated that the genetic similarity between the cultivars in this study is mainly consistent with the trait of flower shape (cut-flower or potted type) (R = 0.701, *p* < 0.01) (Figure S2B).

### 2.8. Transferability and Effectiveness of SSR Primers in Other Species of Brassicaceae Family

**Figure 6:**
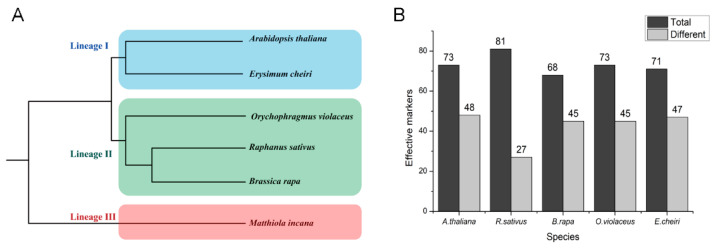
The transferability and effectiveness of SSR primers in five Brassicaceae species. (**A**) The phylogeny of species in Brassicaceae used in this study referenced from Chen et al. (2018). (**B**) The distribution of effective primer pairs in different species. “Total” represents the total number of effective primer pairs in the species; “Different” represents the number of effective primer pairs that have different bands from *M. incana* in the species.

To evaluate the potential transferability and effectiveness of the SSR primers, 96 SSR loci were randomly chosen for amplification on five different species in the Brassicaceae family. These included *Arabidopsis thaliana*, *Raphanus sativus*, *Brassica rapa*, *Orychophragmus violaceus* and *Ersimum cheiri* (Figure 6A), which came from three major lineages [31]. An average of 76.25 (range from 68 to 81) SSR loci were successfully amplified in the five related species (Figure 6B). Clear and stable bands were amplified using 91 of the 96 (94.79%) SSR primer pairs in one or more of the five related species. Most of them exhibited different bands from *M. incana* (Figure 6B), demonstrating the transferability and applicability of the identified SSR primers for *M. incana* in certain relevant Brassicaceae family members.

## 3. Discussion

Stock (*Matthiola incana* (L.) R. Br.) is a popular flowering plant all over the world. However, the lack of molecular markers has limited research into genetic analysis, genome evolution, and marker-assisted selective breeding. In this study, we identified and analyzed genome-wide SSR loci of *M. incana* using preliminary assembled genome data, and developed large-scale SSR primer pairs. Further PCR experiments verified that up to 86.67% of the primers had good amplification. Lastly, high-polymorphism SSR molecular markers were used to analyze and assess the genetic diversity of 40 stock germplasm resources. Besides, these SSR markers are well transferable and effective in other Brassicaceae species. To the best of our knowledge, this is the first report on the large-scale development of SSR molecular markers in *M. incana*, as well as genetic variation in some common cultivars using polymorphic SSR molecular markers.

Currently, the development of SSR molecular markers using NGS data has become a mainstream approach. It has been successfully applied to a variety of plants, such as rice [32], maize [33], watermelon [34], pear [35], cabbage [17], and rapeseed [18]. Using genome-based SSR markers is far more efficient than older methods. The early development methods of SSR markers included the genomic library method [36] and enrichment-based technology [37,38,39]. They have the disadvantages of cumbersome operation, a low success rate and high-cost. As EST (Expressed Sequence Tag) sequences of more and more species became available, they provided a new source of SSR markers [40,41]; however, this approach was limited to information in public databases and the chromosome [42]. With the development of next-generation sequencing (NGS) technology, more and more species have completed whole genome sequencing, which provides an unprecedented amount of data to develop a large number of SSR molecular markers.

In this study, 45,612 genome-wide SSR loci with a density of 23.06 per Mb were identified using a draft genome sequence assembly of *M. incana*. The number of SSR loci was similar to some species in the Brassicaceae family, such as *B. oleracea* (64,546), *B. rapa* (42,656), *B. nigra* (52,117), *R. sativus* (49,605), *B. cretica* (65,262), *E. yunnanense* (53,260), and *C. bursa-pastoris* (46,394) [17]. However, the density (23.06/Mb) of *M. incana* was significantly lower than of *B. oleracea* (132.01/Mb), *B. rapa* (150.13/Mb), *B. nigra* (129.60/Mb), *R. sativus* (123.29/Mb), *B. cretica* (158.20/Mb), *E. yunnanense* (128.22/Mb), and *C. bursa-pastoris* (172.83/Mb) [17]. For this reason, including differences in genome structure among different species, studies have shown that species with larger genomes usually show lower SSR densities [29,43]. For the Brassicaceae species, Xu et al. (2021) also found that the species of *B. rapa*, *C. bursa-pastoris,* and *B. vulgaris,* had larger genomes and a lower density of SSR. For *M. incana*, the genome is large (1977.48 Mb) and each chromosome is very long (ranged from 235.67 Mb to 307.45 Mb), which may lead to low-density. However, some studies showed that the SSR density was not related to genome size [44,45]. Further, there may be differences in identification criteria between different studies. Some studies carried out the SSR motif length which was restrained to 1–6, which was in consistence with mono-nucleotides, di-nucleotides, tri-nucleotides, tetra-nucleotides, penta-nucleotides, and hexa-nucleotides, respectively [17,29], while other studies restrained the SSR motif length to 2–6 bp [30], 2–8 bp [46]. This study identified motifs from di- to hexa-nucleotides, so that numerous mono-nucleotide types were not identified or counted. Finally, different software used in different studies can also lead to different results.

The SSR had abundant motifs, including all types of nucleotide repeats of five motif lengths (2–6 nt). Our results show that the frequency of SSR loci occurrence decreased with increasing motif length (di- to hexa-nucleotides), which is consistent with studies in other species [17,29,30]. Additionally, di-nucleotides repeat units account for 77.18% of all SSR loci. It is worth noting that this ratio was significantly higher than that of many species, including Brassicaceae species, such as *B. rapa* (37.87%), *B. oleracea* (40.94%), *B. nigra* (41.51%), *R. sativus* (44.49%), and *C. bursa-pastoris* (41.35%) [17]. Although the exclusion of mono-nucleotides repeat unit in this study may lead to a higher computational frequency of di-nucleotide repeat unit, we believe that this effect is limited because the frequencies of others (tri- to hexa-nucleotides) are consistent. As a result, the di-nucleotides repeat units in *M. incana* are significantly higher than that in other Brassicaceae species, which may be related to its genome composition, as its genome size is significantly larger than that of other diploid species in Brassicaceae; however, the exact reason is unclear and needs further study. In this study, the base composition of the SSR motifs strongly favored A and T, especially the AT/TA motif. This was not only the dominant di-nucleotides repeat unit (78.58%), but also the most frequent motif in the whole genome, accounting for 60.78% of the total SSR loci (Figure 2D). It has also observed in cucumber [46], eggplant [29], cabbage [17], and other species. It has been reported that AT-rich repeats were common in dicotyledons, but not in monocotyledons [46], and these differences may be explained by the relative nucleotide composition (GC content) of their genomes, as the average GC content of dicots (34.6%) and monocots (43.7%) is different [29,46]. Therefore, the high frequency of AT/TA motif in the *M. incana* genome may be due to the low GC content (about 40%) in the nucleic acid composition.

In the present study, primer pairs were designed and tested for 300 randomly selected SSR loci by electrophoresis, and the effective amplification rate was 86.67% (Table S4). However, only 32 SSR loci were polymorphic, representing 10.67% of the total number of primers. The rate is considered low considering the levels of polymorphism found in other species, such as 43% in rapeseed [18], 50.8% in sea buckthorn [47], and 35% in sago palm [30]. The SSR polymorphism can result from either an elongation or shortening of repeat units. So, the polymorphism of SSR molecular markers is mainly influenced by the length of the SSR, and SSR repeat motif lengths were negatively correlated with the variation rate of SSR lengths [42,48]. The study by Temnykh et al. (2001) on rice showed that when the length of SSR sequence was less than 20 bp, the polymorphism was lower than that of the SSR sequence larger than 20 bp [42]. Shi et al. (2014) found that the SSR marker polymorphism level was positively correlated with the repeat length (r = 0.86) in *Brassica* [49]. Additionally observed were *Cucumis sativus* [46], *Arachis hypogaea* [50], *Hevea brasiliens* [48], and *Punica granatum* [51]. In this study, the majority of the selected SSR motif lengths were less than 40 bp, which may reduce the polymorphism of SSR loci. In addition, factors such as the population numbers of the tested cultivars, and the differences between them, are also responsible for SSR polymorphism in plants. All 40 stock cultivars tested in the current work are horticultural varieties, and the narrow genetic basis may be a significant reason for the lack of polymorphism markers.

The SSR molecular markers have been widely used in crop genetic diversity analysis because of advantages such as their abundant quantity, codominance, simple detection, and stable results [19,20]. In the current work, 22 polymorphic SSR markers successfully assessed the genetic relationships and genetic diversity of 40 stock cultivars. To the best of our knowledge, this is the first report of genetic diversity analysis in stock cultivars; previously, only the infrageneric and intergeneric phylogenetic relationships between Matthiola and other related genera have been reported [14]. We found that these 40 stock cultivars had a close genetic relationship and low genetic diversity (Figure 5), which was consistent with the morphological feature of low phenotypic differences among these varieties (Table S1). The phenotypic differences among the 40 varieties are mainly in plant height and flower color. The consistency between molecular marker clustering and morphological characteristics also confirms the accuracy of the SSR molecular markers we developed in this study.

## 4. Conclusions

In this study, we first report on the development of genome-wide SSR molecular markers for stock (*Matthiola incana* L.) based on NGS genomic data. We identified 45,612 SSR loci and developed 43,540 primer pairs. The success rate of the amplification of the primers by PCR validation was as high as 86.67%. Some identified polymorphic SSR molecular markers have been successfully applied to assess genetic diversity among different stock varieties. Thus, these newly developed potential SSR markers will enrich the resources of SSR markers in *M. incana* for genetic map construction, QTL/gene mapping, marker-assisted selection, and comparative genomic studies, among others. It provides an important genetic tool for the theoretical and applied study of *M. incana*.

## 5. Materials and Methods

### 5.1. Plant Materials

A total of 40 stock cultivars, consisting of commercial cultivars (35 cultivars) and breeding lines (5 cultivars) were used in the potted type and cut-flower type, which have obvious differences in flower shape. Potted type plants have lower plant height and more branches, while cut-flower type plants were opposite (Figure S2A; Table S1). Among these cultivars were some common commercial series in China, such as the ‘Harmony’ series, the ‘Glory’ series, and the ‘Mime’ series, which have very similar phenotypic characteristics (Table S1). For genome sequencing, the pure-line variety ‘Stock Cream White’ was used. The preliminary verification of SSR primer was carried out using ‘Stock Cream White’, ‘Harmony Deep Rose’, ‘Glory Purple’, ‘Xmas Apricot’, ‘Mime Light Pink’, and ‘Iron Blue’ as these varieties come from different series, and contain almost all the variations of the cultivars, such as origin, flower shape, and flower color. All of the 40 cultivars were used for the genetic diversity study (Table S1). These plants were grown in a climate chamber at 23 °C with a 16/8 h photoperiod. The phenotypic data for 40 cultivars were derived from three independent individuals. Genomic DNA was extracted from three plants of each cultivar using the modified CTAB method, then equally mixed and diluted to 50 ng/μL as a DNA template. The DNA quality and concentration were determined by electrophoresis in 1% agarose gel and NanoDrop1000 spectrophotometry (Thermo scientific, Waltham, MA USA). The DNAs were diluted with sterilized ultrapure water and stored at −20 °C.

### 5.2. Library Preparation and Genome Assembly

For Illumina sequencing, we generated ~50× Illumina short reads with an insertion size of 400 bp on the HiSeq 2000 platform (Illumina, San Diego, CA, USA). FastQ (V0.20.0) and FastQC were used to process raw sequencing data to screen and detect reads and overrepresented sequences with poor quality. Chromatin was extracted and digested using standard procedures to construct the HI-C library (PE 150 bp), and the data obtained were used to construct the chromosome-level genome assembly. Kmerfreq was used to analyze Illumina clean reads to estimate genome size. NextDenovo (reads-cutoff: 1 k and seed-cutoff: 32 k) was used to perform de novo assembly. For Hi-C sequence data, after we filtered out low-quality reads and validated paired-end reads, the LACHESIS software [52] was used to cluster, reorder, and orient the contig-scale genome assembly. The Hi-C heatmap was used to manually check the placement and orientation errors apparent in chromosomes. For details of genome sequencing and assembly, refer to Chen et al. (paper in preparation). The sequencing data have been submitted to the public database under PRJNA883049.

### 5.3. Identification of Genome-Wide SSR Loci

Based on our assembled genome of *M. incana*, the assembled contig data (at least 200 bp) were used to identify SSR loci. The SSR motifs were identified using Perl scripts from MISA with default parameters (http://pgrc.ipk-gatersleben.de/misa, accessed on 6 December 2021). A minimum repeat threshold of eight, six, five, five, and five repeats were required for detecting di- to hexa-nucleotides motifs, respectively.

### 5.4. SSR Primer Design and Marker Validation

To design the PCR primers, 200-bp sequences from the flanking regions of the SSR loci were used, with one pair of primers per SSR locus. The Primer 3 software [53] was used to design the SSR primers with the following parameters: the length of primers was in the range of 18–28 bp, with 20 bp as the optimum; the product size range was from 100 to 300 bp; the melting temperature (Tm) was in the range of 55–65 °C, with 60 °C as the optimum and a maximum Tm difference of 1 °C; and with 40–60% primer GC content.

In order to verify these primers, 300 newly developed SSR markers were randomly selected and tested for polymorphism, with six individuals by polymerase chain reaction (PCR), and 6% denaturing polyacrylamide gel electrophoresis (PAGE). The PCR reaction mixture (10 μL) contained 2 μL of template DNA (50 ng/μL), 5 μL of 2 × FineTaq^®^ PCR SuperMix (TransGen, Beijing China), 0.5 μL (10 μM) of each primer solution, and 2 μL of sterilized ddH_2_O. The PCR program followed: 94 °C for 5 min; 13 cycles (94 °C for 30 s, 62 °C for 30 s, 72 °C for 30 s, with a 0.5 °C decrease in annealing temperature at each cycle); 30 cycles (94° for 30 s, 55 °C for 30 s, 72 °C for 30 s); and a final 10 min elongation step at 72 °C. Finally, PCR products were separated on 6% PAGE. We scored markers manually and selected the polymorphic markers for genetic analysis.

### 5.5. Genetic Diversity and Structure Assessment of Stock Varieties

The polymorphism information content (PIC) of markers was calculated as PIC = 1 − ∑(Pi)^2^, where Pi represents the ith allele frequency. The allele number (Na), effective allele number (Ne), and Shannon information index (I) of each primer were calculated by the POPGENE software version 1.32 [54].

## Figures and Tables

**Table 1:** Summary of sequencing and assembly results of *M. incana* genome sequence.

Summary of Genome Assembly	Number
No. of raw reads (Gb)	336
GC percent	40.00%
% Squences with Q30	93.00%
Reads sequences (Mb)	44,459.15
Contig sequence numbers	891
Contigs length (bp)	1,983,607,451
Maximum length of contigs (bp)	41,745,843
N50 of contigs(bp)	11,438,961
Scaffold sequence numbers	343
Scaffold length (bp)	1,983,881,451
Maximum length of scaffold (bp)	357,731,900
N50 of scaffold (bp)	258,639,456
Genome assembly size (Mb)	1977.48
No. of chromosomes	7

**Table 2:** The distribution of SSR loci in the genomic sequences of *M. incana*.

Motifs Length	Number of Loci Identified	Cumulative Length (bp)	Average Length (bp)	Mean of Repeat Number	No. of Motifs	Density (SSR/Mb)
Di-	35,279	725,464	21.76	10.89	6	17.84
Tri-	7319	150,330	20.7	6.92	30	3.70
Tetra-	1264	26,996	21.49	5.37	71	0.64
Penta-	992	28,305	28.56	5.71	105	0.50
Hexa-	758	26,730	35.63	5.94	269	0.38
Total	45,612	957,825	.	.	481	23.06

**Table 3:** The distribution of SSR loci and potential SSR markers on chromosomes of *M. incana*.

Chromosome	Chromosome Length (bp)	SSR loci		Designed Primer Pairs		The Succeed Rate of Primer Designing
		Number	Density (SSR Locus/Mb)	Number	Density (Primer/Mb)	
chr1	235,669,302	5512	23.39	5263	22.33	95.48%
chr2	253,907,236	6524	25.69	6204	24.43	95.10%
chr3	301,678,771	6656	22.06	6355	21.07	95.48%
chr4	361,232,675	8368	23.17	8009	22.17	95.71%
chr5	258,445,478	5693	22.03	5400	20.89	94.85%
chr6	307,454,722	6899	22.44	6601	21.47	95.68%
chr7	259,093,400	5960	23.00	5708	22.03	95.77%
Total	1,977,481,584	45,612	23.07	43,540	22.02	95.46%

**Table 4:** The polymorphism information of markers.

Marker	Na *	Ne *	I *	PIC	Chromosome
MISSR99	2	2.000	0.693	0.500	Chr1
MISSR207	3	2.149	0.826	0.535	Chr1
MISSR209	3	1.256	0.411	0.204	Chr1
MISSR210	3	2.558	1.011	0.609	Chr1
MISSR127	4	3.912	1.375	0.744	Chr2
MISSR221	2	1.663	0.588	0.399	Chr2
MISSR14	4	3.564	1.324	0.719	Chr3
MISSR244	3	2.198	0.856	0.545	Chr3
MISSR227	2	1.190	0.297	0.160	Chr3
MISSR243	2	1.835	0.647	0.455	Chr3
MISSR152	4	3.236	1.260	0.691	Chr4
MISSR155	2	1.724	0.611	0.420	Chr4
MISSR253	4	2.159	0.854	0.537	Chr4
MISSR24	4	2.660	1.108	0.624	Chr5
MISSR264	2	1.882	0.662	0.469	Chr5
MISSR265	3	2.612	1.024	0.617	Chr5
MISSR178	4	3.756	1.353	0.734	Chr6
MISSR271	3	2.632	1.030	0.620	Chr6
MISSR289	5	3.493	1.366	0.714	Chr6
MISSR291	2	1.220	0.325	0.180	Chr6
MISSR5	2	1.551	0.540	0.355	Chr7
MISSR192	4	3.556	1.322	0.719	Chr7
Mean	3.0	2.400	0.886	0.525	

* na = observed number of alleles. * ne = effective number of alleles (Kimura and Crow (1964)). * I = Shannon’s information index (Lewontin (1972)).

## Data Availability

The data analyzed during the current study are available from the corresponding author upon reasonable request.

## Supplementary Materials

The following supporting information can be downloaded at: https://www.mdpi.com/article/10.3390/plants12040748/s1, Figure S1: The electrophoresis results of polymorphism markers; Figure S2: Phenotypic analysis. (A) The flower shape of potted type and cut-flower type, bar= 10 cm. (B) Correlation analysis between the clustering results and phenotypes. * *p*< 0.05, ** *p* < 0.01; Table S1: Stock cultivars used in this study; Table S2: Summary of repeat motifs in SSR loci; Table S3: The designed primers and their information; Table S4: The information of the randomly selected 300 primer pairs used for PCR verification; Table S5: Genetic similarity coefficients of 40 stock cultivars.
