# Supplementary figures and images for: First Report on Development of Genome-Wide Microsatellite Markers for Stock (Matthiola incana L.)
Source: Plants (Basel). 2023 Feb 7;12(4):748. doi: 10.3390/plants12040748 (PMC9965543; doi:10.3390/plants12040748)

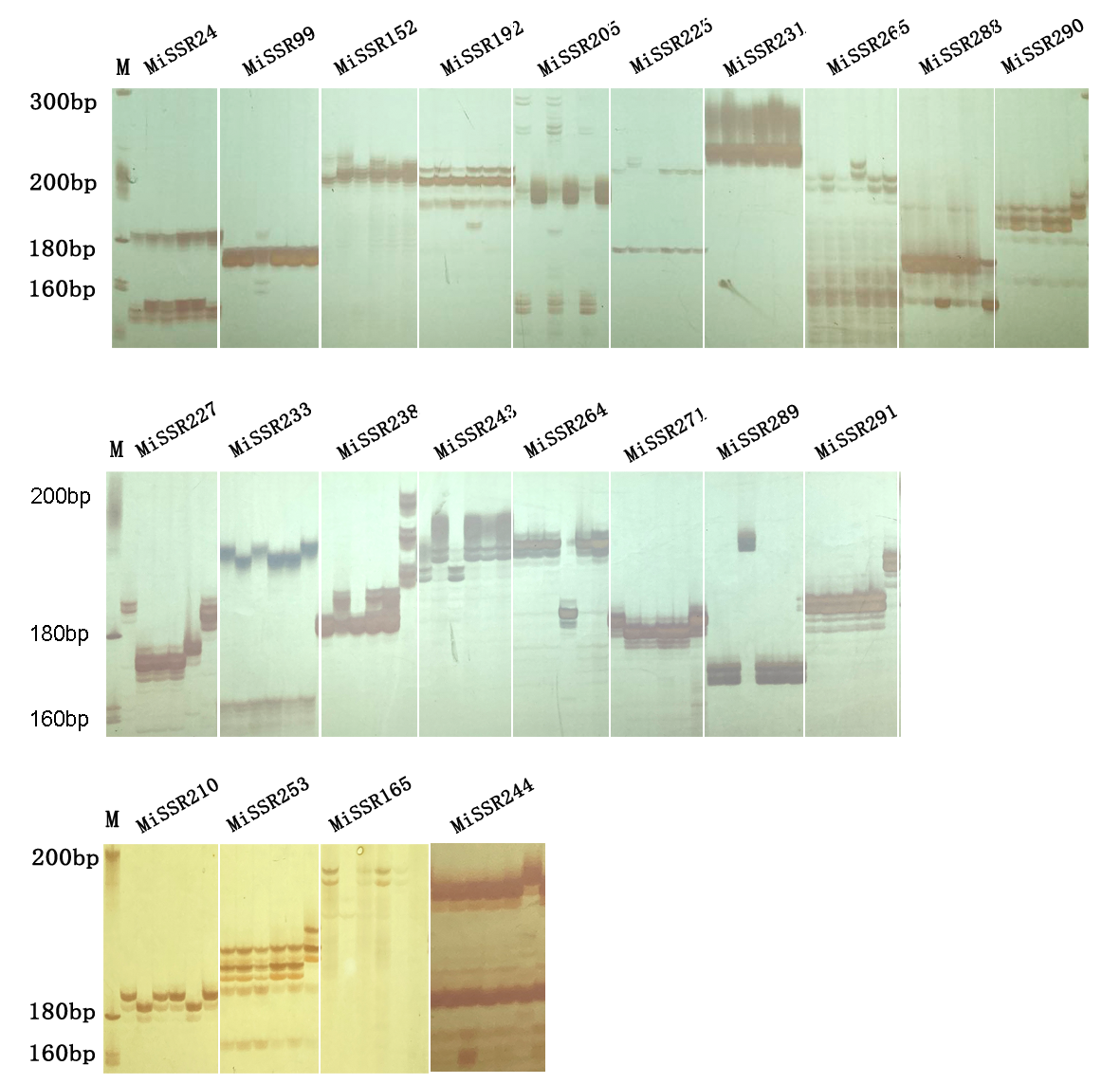

Supplement: Supplementary file 1 [file plants-12-00748-s001.zip › Figure S1.tif]

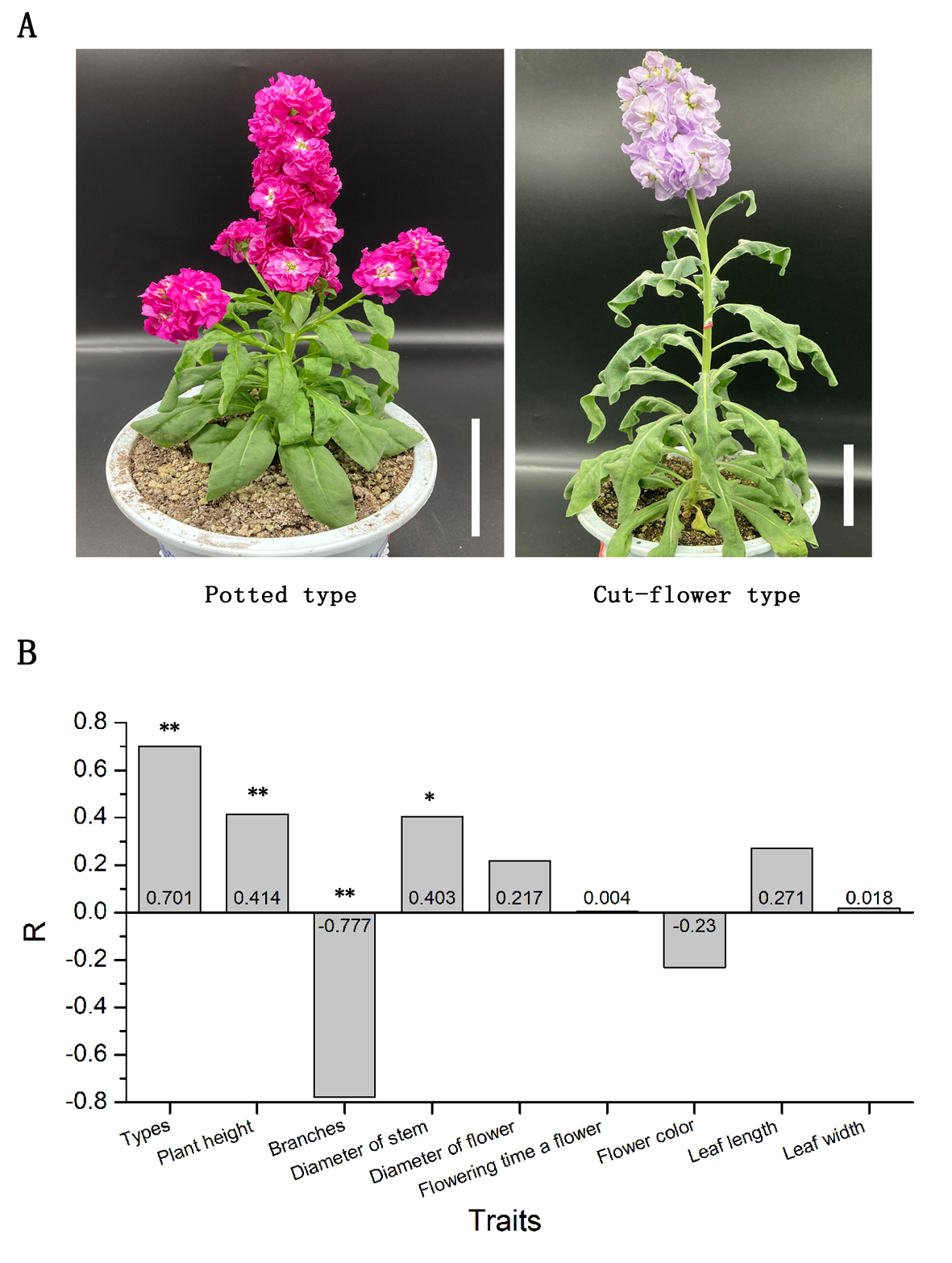

Supplement: Supplementary file 1 [file plants-12-00748-s001.zip › Figure S2.tif]
